# The impact of Verapamil for radial access in diagnostic cerebrovascular angiograms: A retrospective case-control study

**DOI:** 10.1177/15910199231193932

**Published:** 2023-08-13

**Authors:** Dominic Romeo, Mohamed M. Salem, Georgios S. Sioutas, Antonio Corral Tarbay, Jinggang Jenny Ng, Pakinam E. Aboutaleb, Visish M. Srinivasan, Bryan Pukenas, Brian T. Jankowitz, Jan-Karl Burkhardt

**Affiliations:** 1 Department of Neurosurgery, Hospital of the University of Pennsylvania, Penn Medicine, Philadelphia, PA, USA; 2 Department of Neurology, Perelman School of Medicine, 6572 University of Pennsylvania, Philadelphia, PA, USA; 3 Department of Neuroradiology, Hospital of the University of Pennsylvania, Penn Medicine, Philadelphia, PA, USA

**Keywords:** Hives, verapamil, transradial access, diagnostic cerebral catheter angiogram, radial “cocktail”

## Abstract

**Introduction:**

Different combinations of medications are utilized during wrist access for radial artery (RA) or ulnar artery (UA) catheterization in neuroendovascular procedures to preclude vasospasm. These “cocktails” commonly include the calcium channel blocker Verapamil, without established benefit. We analyze outcomes in patients with and without Verapamil in their “cocktail” by using a case-control cohort of our single-center experience.

**Methods:**

A prospective log of consecutive patients who underwent diagnostic cerebral angiograms using RA/UA access was retrospectively reviewed, and patients were grouped into Verapamil and non-Verapamil cohorts. The primary outcomes assessed were the presence of forearm skin rashes (hives) and RA/UA spasms. Our initial management included Verapamil (5 mg) in the cocktail, but Verapamil was removed after we noticed the development of hives in multiple patients immediately following its injection.

**Results:**

A total of 221 patients underwent 241 RA/UA diagnostic cerebral angiograms and were included in our analysis. One hundred and forty-nine patients (61.8%) underwent catheterization with Verapamil and 92 (38.2%) were catheterized without it. Four of the 149 patients in the Verapamil group (2.7%) developed hives during the procedure and were treated with Benadryl (25 mg). Of the 92 patients who did not receive Verapamil, there were zero (0%) cases of hives and one (1.1%) case of vasospasm.

**Conclusion:**

Verapamil in the “cocktail” for wrist access diagnostic cerebral angiograms was associated with periprocedural hives, but not associated with a significant reduction in spasm compared to the non-Verapamil group. Our findings suggest that the administration of prophylactic Verapamil for these procedures may not be necessary.

## Introduction

During the past few years, radial artery (RA) or ulnar artery (UA) access has been increasingly utilized for neuroendovascular procedures, following in the footsteps of interventional cardiology.^1–3^ Several large-scale studies in neurosurgery, neuroradiology, and interventional cardiology have demonstrated that RA/UA access is associated with fewer site complications and higher patient satisfaction than the previously standard trans-femoral approach.^4,5^ However, the diameter of the RA/UA is smaller than that of the femoral artery, theoretically subjecting it to more complications including vasospasm and occlusion, which can lead to arm ischemia or catheter entrapment inside the vessel. To mitigate vasospasm risk, different combinations of medications, commonly referred to as a radial “cocktail,” are usually administered for prophylaxis against intraprocedural vasospasm. These medications include lidocaine, heparin, and nitroglycerine among others, and are intended to decrease vasospasm but can also have side effects including fluctuations in blood pressure and heart rate, local tissue hyperemia, swelling, and hives. Recently, the necessity of some of these medications has been called into question with da Silva et al.^6^ finding that nitroglycerin was not associated with a decrease in RA occlusion rate, despite being commonly included in the RA cocktail. Another commonly used medication without clearly established benefit is Verapamil, a calcium channel blocker that is thought to attenuate vasospasm risk.^7^ Given that the effects of Verapamil in the radial “cocktail” are sparsely reported^8^ in the context of RA/UA neuro-angiography, we seek to assess this association using a retrospective cohort study of our single-center experience. Specifically, this study evaluates whether the use of Verapamil as part of a radial “cocktail” is associated with (a) an increased risk of hives and (b) an increased risk of RA/UA vasospasm compared to diagnostic RA angiograms conducted without Verapamil.

## Methods

### Study population

**Figure 1.**
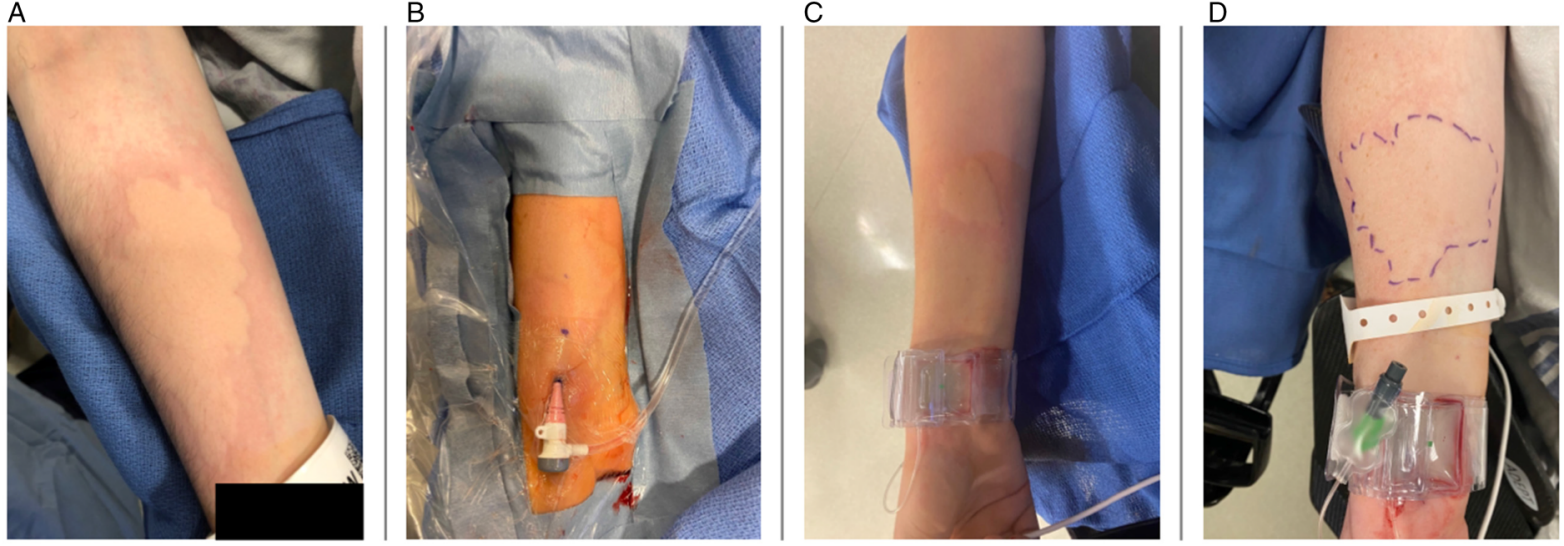
A total of four patients (A–D) developed hives after verapamil administration.

A series of consecutive patients undergoing diagnostic radial angiograms between September 2020 and November 2021 were included in this cohort. The study was approved by the Institutional Review Board, and patient consent was waived due to the retrospective design of the study. The study is in accordance with the Strengthening the Reporting of Observational Studies in Epidemiology guidelines.^9^ Patients who received Verapamil in their medication cocktail were classified as “cases” and patients without Verapamil were “controls.” We limited the analysis to diagnostic angiograms, excluding treatment procedures to avoid potential confounding factors related to the complexity of larger sheaths/catheters and prolonged procedures due to interventions. Basic clinical characteristics of patients as well as data on primary and secondary outcomes were collected. The primary outcomes assessed were the presence of hives and RA spasms. Hive development was defined as the appearance of urticarial lesions that are circumscribed, erythematous, and raised wheals/plaques, often with central pallor (Figure 1A to D). The skin reaction was observed either during or most commonly right after the procedure when the drape was removed from the forearm. Arm pain was assessed post-procedure, and some patients with skin reactions had pain in the area of the lesions. Clinical vasospasm was defined by decreased oxygen levels on pulse oximetry (<80), significant arm pain with or without catheter movement, or a catheter unable to be removed from the RA. Secondary outcomes included procedure length, femoral conversion rates, and procedural side effects that might be attributed to Verapamil administration (e.g. local access site complications such as bleeding, pseudoaneurysms, and arteriovenous fistula). Bleeding events were defined according to the global use of strategies to open occluded arteries bleeding wherein clinical criteria in scales such as the thrombolysis in myocardial infarction and global use of strategies to open occluded coronary arteries were found to be the best way to assess for bleeding complications.^10,11^

### Procedural protocol/radial “cocktail”

After locally anesthetizing the skin overlying the distal RA with lidocaine or bupivacaine, a small ultrasound-guided incision was made, and a 5F or 6F radial short sheath (Merit) was placed in the vessel using standard technique. Next a radial “cocktail” including 2000 U Heparin, 200 mcg of nitroglycerin, and—in the Verapamil group (September 2020 to May 2021)—5 mg of Verapamil was then slowly administered through the radial sheath. Systemic blood pressure was monitored for 2 min, and an RA angiography was then performed over the forearm. For the diagnostic cerebral angiogram, a 0.035″ Glidewire (Terumo) and a 5 French SIM-2 sidewinder glide catheter (Terumo) or 5/6 French Sim2 Select catheter (Penumbra) were used. Distal RA access was defined as the RA in the anatomic snuffbox and proximal was defined as the region of the RA 2 cm proximal to the radial styloid process. Proximal or distal RA access was chosen due to operator preference and vessel size, whereas UA access was only chosen if RA was too small or occluded. Allen's test was not performed; an arm angiogram confirmed patent forearm/hand blood supply at the beginning of the angiogram and continued pulse oximetry was performed at the hand/finger of access to ensure sufficient blood supply. Tortuous anatomy was defined as the presence of unfavorable anatomy including anatomical variations within the arm such as radial arteries with an ectopic brachial origin, radioulnar loops, bifurcated RA, or severe tortuosity of the right subclavian artery or aortic arch. Procedural time was recorded as the period from when the sheath was inserted to the moment it was withdrawn.

### Statistical analysis

Continuous variables are presented as a mean ± standard deviation or median/interquartile range (IQR) based on data normality, while the categorical variables are expressed as proportions. T-test/Mann–Whitney *U*-test was applied to compare the continuous variables as appropriate while the chi-square test (χ^2^) was used to compare the categorical variables. A two-tailed probability level of <0.05 was considered statistically significant. All statistical analyses were performed using STATA version 15.0 (StataCorp).

## Results

### Baseline characteristics

A total of 221 patients (median age 59.2; IQR: 47.7–68.9; 69.2% females) undergoing 241 radial diagnostic neuro-angiography procedures were included in this analysis. History of hypertension was present in 53.9% of the patients, history of any calcium channel blocker use was present in 55.7%, and the median body mass index (BMI) of patients included in our study was 27.8 kg/m^2^ (IQR: 24.4–3 3.5) (Table 1).

**Table 1.** Baseline descriptive statistics.

Number of patients	*n* = 221
Gender	
Male	68 (30.8%)
Female	153 (69.2%)
Age (years: median; IQR)	59.2 (47.7–68.9)
Race	
Caucasian	119 (53.9%)
African American	72 (32.6%)
Asian	14 (6.3%)
Hispanic	7 (3.2%)
Other/unknown	9 (4.1%)
Smoking status	
Current smokers	25 (11.3%)
Former smokers	6 (2.7%)
Non-smokers	190 (86%)
Hypertension	119 (53.9%)
History of calcium channel blocker use*	
Any calcium channel blocker	123 (55.7%)
Dihydropyridine and/or non-dihydropyridine	100 (45.2%)
Gabapentinoids	33 (14.9%)
BMI (median: IQR)	27.8 (24.2–33.5)

BMI: body mass index; IQR: interquartile range.

*Prior or current use, any dose or duration.

### Procedural characteristics

All 221 patients underwent 241 ultrasound-guided wrist catheterizations. Of the 241 angiograms, 20 were patients who underwent more than one angiogram; in all but one, the access route was on the same side, and none of these patients developed hives. In most procedures (97.5%), the right RA was catheterized and proximal RA was the most common point of access (66%), followed by distal RA and UA (24.1% and 6.6%, respectively). In 3.3% of the patients, the procedure was converted to femoral access due to vessel anatomy. Tortious RA anatomy was noted in 4.6% of the procedures and the median RA diameter at the point of access was 2.4 mm (IQR: 2–2.7). Most catheterization attempts were successful on the initial cannulation attempt (93.4%), and 16 (6.6%) were unsuccessful. Of the 16 unsuccessful on their initial attempt, 15 (6.2% of the total) were successful on the subsequent attempt, and one (0.4% of the total) was successful on the third attempt. Monitored anesthesia care was used in 79.7% of procedures, while general anesthesia was used in 20.3%. Five French catheters were used in 232 (96.3%) patients, and six French catheters were used in four (1.7%) patients. The average procedure length was 11.6 min (IQR: 8.6–17). A total of four patients (1.8%) developed hives and one patient (0.4%) had a severe RA spasm. Procedural characteristics are summarized in Table 2.

**Table 2.** Procedural characteristics.

Procedures (wrist diagnostic angiograms)	*N* = 241
Ultrasound-guided wrist catheterization	241 (100%)
Side	
Right	235 (97.5%)
Left	6 (2.5%)
Point of access	
Proximal radial	159 (66%)
Distal radial	58 (24.1%)
Ulnar	16 (6.6%)
Conversion to femoral	8 (3.3%)
Tortuous radial artery anatomy (e.g. loops)	11 (4.6%)
Radial artery diameter (at point of access) (mm, median; IQR)	2.4 (2–2.7)
Non-successful initial catheterization	16 (6.6%)
Catheterization attempts number	
1	225 (93.4%)
2	15 (6.2%)
3	1 (0.4%)
Anesthesia	
Monitored anesthesia care/conscious sedation	192 (79.7%)
General anesthesia	49 (20.3%)
Access sheath*	
5Fr	232 (96.3%)
6Fr	4 (1.7%)
Procedure length (min, median; IQR)	11.6 (8.6–17)
Radial cocktail	
Including verapamil	149 (61.8%)
Without verapamil	92 (38.2%)
Procedural outcomes	
Development of hives	4 (1.7%)
Severe RA spasm	1 (0.4%)

IQR: interquartile range; RA: radial artery.

*Missing for 5.

### Comparison between the Verapamil and non-Verapamil groups

The procedures including Verapamil in the medication cocktail were summarized and compared to the non-Verapamil group: out of 241 procedures, 61.8% of the patients received Verapamil while 38.2% did not. There was no significant difference in terms of age and gender distribution between the groups (*p* = 0.94 and *p* = 0.88, respectively) and BMI distribution did not differ significantly between the groups (median 27.7 (IQR: 24.2–33.4) kg/m^2^ in the Verapamil vs. median: 28.3 (IQR: 24.3–33.7) kg/m^2^ in the non-Verapamil group; *p* = 0.61). Similarly, the RA diameter at the puncture site was similar between the groups (2.3 (IQR: 2–2.6) mm vs. 2.4 (IQR: 2.1–2.8) mm; *p* = 0.24). The proportion of those undergoing proximal RA catheterization was higher in the non-Verapamil group (80.5% vs. 70.6%); however, it did not reach statistical significance (*p* = 0.1). Positive smoking history was significantly higher in the Verapamil group (18.8% vs. 8.7%; *p* = 0.033). Similarly, Verapamil was used at a higher rate in cases when initial cannulation failed (10.1.% vs. 1.1%; *p* = 0.007). All left RA procedures utilized Verapamil (p = 0.05). Significantly longer procedure times were noted in the Verapamil group (median 12.7 (IQR: 8.9–18.1) min vs. median 10.4 (IQR: 7.913) min; *p* = 0.002). However, there were no significant differences in terms of femoral conversion rates between the groups (2% in the Verapamil group vs. 5.4% in the non-Verapamil group; *p* = 0.2). The development of hives during RA catheterization occurred exclusively in the Verapamil group (2.7% vs. 0%; *p* = 0.1), while RA spasms occurred in one case in the non-Verapamil group (1.1% vs. 0%; *p* = 0.23). There were no procedural complications encountered in either group (Table 3). In a subgroup sensitivity analysis in cases with only one catheterization attempt, the Verapamil and non-Verapamil groups did not significantly differ in terms of RA/UA spasm (0.0% vs. 1.1%, respectively; *p* = 0.404) and hives (3.0% vs. 0.0%, respectively; *p* = 0.124)

**Table 3.** Comparison between Verapamil and non-Verapamil groups.

	Verapamil group	No Verapamil group	*p* value
	149 (61.8%)	92 (38.2%)	
Age (years, median; IQR)	59.8 (47.6–68.8)	58.5 (47.9–69.6)	0.94
Female gender	105 (70.5%)	64 (69.6%)	0.88
Caucasian race	48 (32.2%)	28 (30.4%)	0.77
Positive smoking history	28 (18.8%)	8 (8.7%)	0.033
Hypertension	83 (55.7%)	47 (51.1%)	0.49
History of calcium channel blocker use*			
Any calcium channel blocker	87 (58.4%)	51 (55.4%)	0.65
Dihydropyridine and/or non-dihydropyridine	69 (46.3%)	44 (47.8%)	0.82
Gabapentinoids	26 (17.5%)	12 (13.0%)	0.36
Body mass index (median; IQR)	27.7 (24.2–33.4)	28.3 (24.3–33.7)	0.61
Access sheath size			
5Fr	145 (97.3%)	87 (98.8%)	0.27
6Fr	4 (2.7%)	1 (1.2%)	
Proximal versus distal RA catheterization			
Proximal	101 (70.6%)	66 (80.5%)	0.1
Distal	42 (29.4%)	16 (19.5%)	
Left RA access	6 (100%)	0 (0%)	0.05
RA diameter (at the site of puncture) (mm, median; IQR)	2.3 (2–2.6)	2.4 (2.1–2.8)	0.24
Failure of initial catheterization attempt	15 (10.1%)	1 (1.1%)	0.007
Length of procedure (min, median; IQR)	12.7 (8.9–18.1)	10.4 (7.9–13)	0.002
Femoral conversion	3 (2%)	5 (5.4%)	0.2
Presence of hives after the procedure	4 (2.7%)	0 (0%)	0.1
Severe RA spasm	0 (0%)	1 (1.1%)	0.23

IQR: interquartile range; RA: radial artery.

*Prior or current use, any dose or duration. p value < 0.05 (denotes statistical significance).

### Hives versus RA spasm demographics

A total of four patients developed hives (Figure 1A to D). All four patients (100%) were female (*p* = 0.19), without any history of calcium channel blocker use. The patients who developed hives were significantly younger than the rest of the cohort (median age 27.6 (IQR: 25–33) years vs. median 59.8 (IQR: 47.9–69.3) years; *p* = 0.003) and the median BMI was lower in the hives group versus the rest of the cohort (23.1 (IQR: 20.3–27.5) kg/m^2^ vs. 28 (IQR: 24.3–33.5) kg/m^2^; *p* = 0.07, Table 4). In all four patients, the hives resolved within 2 h following the angiogram without further negative sequelae after administering 25 mg diphenhydramine. The patient who developed severe RA spasm was a female with a BMI of 33.3 with a small RA diameter of 1.6 mm at the site of the puncture using a 5F radial sheath/5F catheter, eventually requiring femoral conversion. Although RA/UA spasms and hives were present only in cases without general anesthesia (0.5% and 2.1%, respectively), this difference was not significant compared to general anesthesia (*p* = 0.621 and *p* = 0.384, respectively).

**Table 4.** Comparison of hives versus non-hives group.

	Hives group	No hives group	*p* value
	4 (2.8%)	217 (38.2%)	
Age (years, median; IQR)	27.6 (25–33)	59.8 (47.9–69.3)	**0**.**003**
Female gender	4 (100%)	165 (69.6%)	0.19
Body mass index (median; IQR)	23.1 (20.3–27.5)	28 (24.3–33.5)	0.07

IQR: interquartile range. p value < 0.05 (denotes statistical significance).

## Discussion

Over the past two decades, RA diagnostic angiograms have developed into the preferred method of access for cardiac interventionalists, and a similar trend has manifested itself among neurointerventionalists.^12^ Many experts hold that the prophylactic administration of intra-arterial vasodilators is necessary.^13,14^ While some studies examine the relative advantages of vasodilating agents such as phentolamine and isosorbide dinitrate, as many as 75.3% of interventionalists use the L-type calcium channel blocker Verapamil in their “radial cocktail,” without clearly established benefit.^8,15–18^ There are also geographical discrepancies in the use of vasodilators for prophylaxis in RA spasm, with as many as 72.2% of Japanese interventionalists not using any medication for spasm prophylaxis, suggesting that the use of Verapamil may not be essential in RA cannulation procedures.^19^

The use of prophylactic Verapamil in the RA cocktail is not without risks. Indeed, adverse side effects of Verapamil including bradycardia are reported in the cardiac and neurologic literature, particularly when Verapamil is used in combination with other medications, in patients with medical comorbidities such as kidney disease, at high doses, or over extended periods of time.^20,21^ There are reports in which Verapamil is used at low doses but still results in significant bradycardia.^22^ Additionally, side effects of Verapamil such as skin rashes and feet edema have been reported, though not in the context of neuroendovascular interventions.^23^ Taking into consideration the various risks associated with using Verapamil, some suggest that the prophylactic use of Verapamil in RA procedures may not be necessary.^19^

A randomized, double-blind trial in the cardiac literature by Hizoh et al.^19^ scrutinized the need for verapamil administration in RA interventional cardiac procedures. These authors compared the rate of access site conversions, procedural times, contrast volume, and subjective pain in the Verapamil and placebo groups. They found that the rate of access site conversions, procedural times, contrast volume, and pain score were similar in both groups, suggesting that Verapamil prophylaxis may be not needed in RA catheterization procedures.^19^ They used the same dosage of Verapamil as in this study, but did not include nitroglycerin, and used a wider range of radial sheaths (4-7F vs. 5-6F).

To our knowledge, this is the first study to analyze the safety and patient outcomes associated with the use of Verapamil compared to a group of patients without it in RA diagnostic angiograms in the neurointerventional literature. This cohort study includes 221 consecutive cases performed via RA or UA access. Overall, there were a total of four (2.7%) cases of temporary hives that were successfully treated with 25 mg diphenhydramine, and all of them happened in the Verapamil subgroup. No hives developed in patients who underwent RA diagnostic angiograms without Verapamil in their radial cocktail. Additionally, there were no vasospasms in the Verapamil group and one instance in the non-Verapamil group (*p* = 0.23), likely related to the smaller vessel diameter and high BMI precluding successful cannulation rather than a lack-of-Verapamil effect. Studies on transradial coronary procedures have associated high BMI and small radial diameter with spasms.^24,25^ However, other topical anesthetics such as lidocaine can cause hives too.^26^

Though limited by the small number of events given that we ceased using Verapamil after noticing these adverse effects, hives seemed to occur in a younger subset of all-female patients (median age 27.6 vs. 59.8 years in the cohort), with a smaller BMI compared to the rest of the cohort (23.1 kg/m^2^ vs. 28 kg/m^2^). This may suggest that lacking subcutaneous fat tissue could increase the likelihood of developing a skin rash due to the local Verapamil effect in the forearm. The location of all four instances of hives supports this theory since all cases of hives developed near the tip of the radial sheath in the proximal one-third of the forearm. However, further evidence from larger studies is needed to draw additional conclusions.

We also found significantly longer procedure times in the Verapamil group (median 12.7 min vs. median 10.4 min; *p* = 0.002) compared to the non-verapamil group as well as no significant difference in femoral conversion rates between the groups (2% in the Verapamil group vs. 5.4% in the non-Verapamil group; *p* = 0.2). Our data also show that the Verapamil group experienced a higher rate of failure of the initial catheterization attempt than the non-Verapamil group (10.1% vs. 1.1%, *p* = 0.007; Table 3), likely leading to longer procedure times in the Verapamil group. Failure of initial catheterization was likely unrelated to Verapamil as it is administered after initial catheterization and instead likely reflects the operators’ anticipation of vasospasm risk given that multiple catheterization attempts are a known risk for RA spasm.^27^

Patients undergoing neuroendovascular procedures are often medically complex and possess multiple cardiovascular risk factors such as hypertension, diabetes, and hyperlipidemia.^28^ To mitigate the risk associated with these conditions, they are increasingly burdened by higher medication loads and thus possess polypharmacy-associated complications. Therefore, removing Verapamil from the radial cocktail decreases polypharmacy complications such as neurological, gastrointestinal, and psychiatric sequelae. Adding to the risk and benefit equation, hives and RA/UA spasm rates did not differ between the Verapamil and non-Verapamil groups.

Limitations of this study include the low number of events and limited sample size (221 total RA diagnostic angiograms) with low instances of both hives (only four cases) and RA spasms (only one severe case), which precludes a multivariable analysis controlling for other confounders and likely precludes reaching statistical significance for some comparisons. Another limitation is related to the study design as a retrospective analysis of prospectively collected cases and the intrinsic biases associated with this study design. Finally, changes in blood pressure and heart rate during verapamil administration were impossible to be recorded due to the retrospective nature of this study.

## Conclusion

In this study, Verapamil as part of the radial “cocktail” for radial access diagnostic cerebral angiograms was associated with periprocedural hives, but not associated with a significant reduction in spasm or femoral conversion rates when compared to the non-Verapamil group. Our findings suggest that routine administration of prophylactic Verapamil as part of the radial “cocktail” for radial access diagnostic cerebral angiograms may not be needed.
